# Antioxidant, Anti-Lipoxygenase and Cytotoxic Activity of *Leptadenia pyrotechnica *(Forssk.) Decne Polyphenolic Constituents

**DOI:** 10.3390/molecules16097510

**Published:** 2011-09-05

**Authors:** Mohammad A. Khasawneh, Hanan M. Elwy, Alaaeldin A. Hamza, Nael M. Fawzi, Ahmed H. Hassan

**Affiliations:** 1 Department of Chemistry, Faculty of Science U.A.E. University, Al-Ain, P.O. Box 17551, UAE; 2 National Organization of Drug Control and Research, 6 Abu Hazem St., Giza, Egypt; 3 Department of Biology, Faculty of Science U.A.E. University, Al-Ain, P.O. Box 17551, UAE; 4 Department of Biochemistry, Faculty of Medicine and Health Sciences, U.A.E. University, Al-Ain, P.O. Box 17666, UAE

**Keywords:** *Leptadenia pyrotechnica*, total phenol, antioxidant, anti-inflammatory, cytotoxicity

## Abstract

*Leptadenia pyrotechnica* Forssk is a traditional medicinal herb used for treatment of inflammatory diseases and cancer. In this research, the aqueous ethanolic crude extract of *Leptadenia pyrotechnica* aerial parts, along with its ethyl acetate, *n*-butanol and water partitioning fractions were evaluated for their antioxidant capacity, polyphenolic content, anti-inflammatory and anti-cancer properties. The total antioxidant capacity was estimated by the FRAP, DPPH, ABTS and β-carotene bleaching assays.The ethyl acetate fraction exhibited the highest polyphenolic content (252.27 mg gallic acid/g) and the best antioxidant activity (1.2, 0.57, 0.45 mmol ascorbic acid equivalent/g in the FRAP, ABTS and DPPH assays, respectively). Furthermore, the same extract showed appreciable anti-inflammatory via lipoxygenase (LOX) inhibitory activity (IC_50_ = 1.41 µg/mL). Moreover, the ethyl acetate fraction also showed the strongest cytotoxic effect (IC_50_ = 43.16 µg/mL) against MCF-7 human breast cancer cell line. These results suggest that this plant may be considered an interesting source of compounds with antioxidant, anti-inflammatory and anti-cancer properties for therapeutic, nutraceutical and functional food applications.

## 1. Introduction

Plants still represent a large source of structurally novel compounds that might serve as leads for the development of novel drugs, nutraceuticals and functional foods. There is an increasing interest in naturally occurring antioxidants to replace synthetic counterparts used for food preservation, flavoring, and cosmetics, as well as in health promotion. Polyphenolic substances, which are largely found in most plants, exhibit a wide range of biological effects including anti-inflammatory, anti-microbial and anti-cancer effects [1,2].

*Leptadenia pyrotechnica* (Forssk.) Decne, belonging to the family of Asclepiadaceae, is widespread in tropical Africa, Asia and the Mediterranean region and in the sandy plains in the Western Gulf countries. Currently, it is being cultivated in forests, farms and on the sides of roads [3,4]. It is commonly used traditionally for the treatment of a variety of inflammation-related disease including rheumatism, asthma, wound healing and tumors [5]. The leaves and bark of the plant are used in folk medicine in Mali to prepare antispasmodic, anti-inflammatory and antibacterial remedies [6]. Recently, it has been reported to have hypolipidemic and anti-atherosclerotic effects [7] as well as antitumor activity [8]. To date, this anti-inflammatory property is well documented and it would be interesting to learn whether this activity is mediated by inhibition of lipoxygenase activity. Lipoxygenases are the key enzymes in the biosynthesis of leukotrienes that play an important role in several inflammation-related diseases such as arthritis, asthma, cancer and allergic diseases [9]. *Leptadenia pyrotechnica* is known to be a source of novel components such as pregnane glycocides and flavonoid glycosides [6,8], which may work as antioxidants against reactive oxygen species (ROS) responsible for the oxidative damage processes [10]. 

This study was focused on the quantitative determination of the phenolic content, antioxidant, anti-lipoxygenase and anti-cancer activities of the aqueous ethanol extract of *Leptadenia pyrotechnica* Forssk along with its ethyl acetate, *n*-butanol and water partitioning fractions. The antioxidant potential was evaluated in comparison with the scavenging power of the two stable nitrogen–centered radicals, 1,1-diphenyl-2-picrylhydrazyl (DPPH·) and 2,2-azino-bis(3-ethylbenzothiazoline-6-sulfonate) radical (ABTS·^+^). The reducing power of antioxidants was evaluated by the ferric reducing antioxidant power (FRAP) assay as well as anti-bleaching of β-carotene activity. The anti-inflammatory effect was also examined via lipoxygenase inhibitory activity. Finally, the cytotoxic activity was evaluated using the *in vitro* MTT (3-(4,5-dimethylthiazol-2-yl)-2,5-diphenylformazan) assay on the MCF-7 human breast cancer cell line.

## 2. Results and Discussion

### 2.1. Identification of Main Polyphenolic Compounds

HPLC analysis revealed the presence of various compounds in *Leptadenia pyrotechnica e*xtracts (Table 1). Quantitative and qualitative identification of the various plant extracts were performed against authentic standards of six probable polyphenolic compounds (gallic acid, vanillic acid, caffeic acid, epicatechin, *trans*-cinnamic acid, and quercetin-3-*β*-D-glucoside). The crude ethanol extract was found to contain five of these six compounds, with epicatechin, vanillic acid, quercetin-3-β-D-glucoside being the highest in concentration (8.76, 7.03, 4.53 mg/g extract, respectively). *trans*-cinnamic acid was not detected in the crude extract, probably because it fell below the detection limit of the method. On the other hand, all six previously mentioned polyphenolic compounds were detected in the ethyl acetate fraction, with quercetin-3-*β*-D-glucoside, vanillic acid and epicatechin being the highest in concentration (16.72, 8.15 and 7.64 mg/g extract, respectively. The *n*-butanol fraction was found to contain lower concentrations of vanillic acid, epicatechin and quercetin-3-β-D-glucoside (3.42, 3.24 and 1.56 mg/g extract, respectively) as the major components. Finally, only vanillic acid and gallic acid were detected in the water fraction in minute concentrations (1.20 and 0.42 and mg/g, respectively). From these results we see that the polyphenolic compounds were concentrated in the ethyl acetate fraction as compared with the *n*-butanol and water fractions.

**Table 1:** Contents of main polyphenolic compounds of ethanol extract and soluble fractions from *Leptadenia pyrotechnica*.

Fraction	Retention time (min)	Compound identified	Contents (mg/g extract)
ethanol	8.84	gallic acid	0.95 ^b, c, d^
	20.71	vanillic acid	7.03 ^b, c, d^
	22.79	caffeic acid	0.48 ^b, c^
	27.20	epicatechin	8.76 ^b, c^
	73.54	quercetin-3-*β*-D-glucoside	4.53 ^b, c^
ethyl acetate	8.93	gallic acid	0.23 ^a, b, c^
	20.49	vanillic acid	8.10 ^a, b, c^
	22.62	caffeic acid	0.78 ^a, c^
	26.18	epicatechin	7.64^ a, c^
	67.71	*trans*-cinnamic acid	1.31
	73.35	quercetin-3-*β*-D-glucoside	16.72^ a, c^
n-butanol	22.07	gallic acid	0.17 ^a, b, d^
	73.04	vanillic acid	3.42^ a, b, d^
	22.07	caffeic acid	0.23 ^a, b^
	26.18	epicatechin	3.25^ a, b^
	73.59	quercetin-3-*β*-D-glucoside	1.56^ a, b^
water	8.84	gallic acid	0.42 ^a, b, c^
	20.71	vanillic acid	1.20 ^a, b, c^

### 2.2. Antioxidant, Free Radical Scavenging Activity and Total Phenolic Content

Due to the complex nature of the different phytochemical classes present in plants, the antioxidant capacities of plant extracts cannot be evaluated accurately using a single method. In the present work, the FRAP, ABTS, DPPH· and β-carotene assays were used to assess the antioxidant activities of *Leptadenia pyrotechnica* extracts. The results of the four assays are summarized in Table 2. The crude ethanol extract exhibited modest antioxidant properties (expressed by the TAC or IC_50_ values) in the four aforementioned assays. The ethyl acetate fraction of *Leptadenia pyrotechnica*, however, exhibited significantly improved antioxidant properties in the four assays. Both of *n*-butanol and water fractions exhibited the lowest antioxidant properties. These results suggest that the antioxidant compounds extracted from *Leptadenia pyrotechnica* are more concentrated in the ethyl acetate fraction. 

**Table 2:** Total antioxidant activity of ethanol extract and soluble fractions from *Leptadenia pyrotechnica* expressed as ascorbic acid equivalents (mmol/g of dry extract). BHT was used as positive control.

Fraction	FRAP assay	ABTS Assay		DPPH Assay		ß -Carotene Assay
	TAC (mmol/g)	TAC (mmol/g)	IC_50__(_µg/mL)	TAC (mmol/g)	IC_50_ µg/mL	IC_50_ (µg/mL)
ethanol	0.24 ± 0.01^ b, c, d^	0.45 ± 0.01 ^b, c, d, e^	71.48 ± 0.04^ c, d^	0.37 ± 0.01^ c, d^	95.99 ± 0.05^ c, d^	86.6 ± 0.40^ b, c, d, e^
ethyl acetate	1.21 ± 0.02^ a, c, d^	0.57 ± 0.001 ^a, c, d, e^	56.68 ± 0.04^ c, d^	0.45 ± 0.04^ c, d^	70.75 ± 1.35^ c, d^	51.52 ± 1.5^ a, c, d, e^
*n*-butanol	0.08 ± 0.001 ^a, b^	0.23 ± 0.01 ^a, b, d, e^	152.01 ± 12.19^ a, b, e^	0.06 ± 0.01^ a, b, e^	546.6 ± 6.7^ a, b, d, e^	135.5 ± 1.5^ a, b, d, e^
water	0.07 ± 0.001 ^a, b^	0.19 ± 0.003^ a, b, c, e^	182.04 ± 7.21^ a, b, e^	0.05 ± 0.01^ a, b, e^	701.96 ± 43.2^ a, b, c, e^	174.5 ± 4.05^ a, b, c, e^
BHT	-	0.42 ± 0.004^ a, b, c, d^	77.85 ± 0.85^ c, d^	0.43 ± 0.01^ c, d^	87.98 ± 7.69^ c, d^	36.85 ± 1.15^ a, b, c, d^

Values are means ± SE of three experiments. Data with letters are significantly different (p < 0.05).

A similar trend was also observed in the values of the total phenolic content measured using the Folin-Ciocalteu reagent assay (Table 3). The crude ethanol extract was found to have a total phenolics content of 69 mg gallic acid equivalent/g). As expected, the ethyl acetate fraction exhibited the highest amount of total phenolics (252.27 mg gallic acid equivalent/g). *n*-Butanol and water fractions, on the other hand, exhibited significantly smaller total phenolic contents (19.14 and 17.76 mg gallic acid/g, respectively).

The results of the antioxidant capacity and total phenolic content are supported by the results of the HPLC analysis discussed in section 2.1 above. Therefore, the better biological properties observed for ethyl acetate fraction could be presumably attributed to its major phenolic compounds such as quercetin-3-*β*-D-glucoside, epicatechin and vanillic acid.

The linear correlation coefficients between the antioxidant capacity (measured by FRAP, DPPH, ABTS and β-carotene assays and total phenolic content of the four fractions are summarized in Table 4. Values for R^2^ varied from 0.99 for FRAP assay to 0.85 DPPH assay, which signals that the presence of phenolic compounds contributes significantly to the antioxidant activity of the tested extracts, especially in the FRAP assay. This result is in agreement with previous reports that have demonstrated a liner correlation between the total phenolic content and the reducing antioxidant capacity of some plant extracts [11,12].

**Table 3:** Total phenolic content of ethanol extract and soluble fractions from *Leptadenia pyrotechnica* expressed as gallic acid equivalents (mg/g of dry extract).

Fraction	Yield (%)	Total Phenolic Content * ( mg/g)
ethanol Extract	6%	75.69 ± 4.39^ b, c, d^
ethyl acetate	0.6%	252.27 ± 2.84^ a, c, d^
n-butanol	9%	19.14 ± 0.94^ a, b^
water	4%	17.76 ± 1.50^ a, b^

* Values are means ± SE of three experiments. Data with on letters are significantly different (p < 0.05).

**Table 4:** Linear correlations between the amount of total phenolic content and antioxidant activities of ethanol extract and soluble fractions from *Leptadenia pyrotechnica*.

Assay	Correlation (R^2^)	Significance
FRAP activity	0.99	P < 0.001
ABTS· scavenging activity	0.90	P < 0.01
DPPH· scavenging activity	0.85	P < 0.01
β-carotene bleaching inhibition	0.87	P < 0.01

It is noteworthy that the ethyl acetate fraction exhibited relatively better antioxidant properties than the commonly used antioxidant BHT in both the ABTS and DPPH assays and only slightly less in the β–carotene assay. It is also interesting to note that the ABTS radical scavenging ability of the ethyl acetate fraction was much higher than those values reported for other plants such as peanuts (81.3 µmol/g), pistachios (75.9 µmol/g) and almonds (25.4 µmol/g) [13]. Additionally, the DPPH radical scavenging ability of the ethyl acetate fraction was relatively higher than those reported for other common plants such as rosemary (IC_50_ = 200 µg /mL), sage (IC_50 _= 400 µg /mL) and thyme (IC_50_ = 470 µg /mL) [14] and fennel (IC_50_ = 148 µg /mL), cress (IC_50_ = 148 µg /mL) and chicory (IC_50_ = 76 µg /mL) [15]. Finally, the inhibition ability of bleaching of β-carotene of the ethyl acetate and ethanol fractions was much higher than those reported for other common plants such as mint (IC_50_ > 100 µg /mL) and radish (IC_50_ > 100 µg /mL) [16] and chicory (IC_50_ > 100 µg /mL) [15].

### 2.3. LOX Inhibition Assay

**Figure 1:**
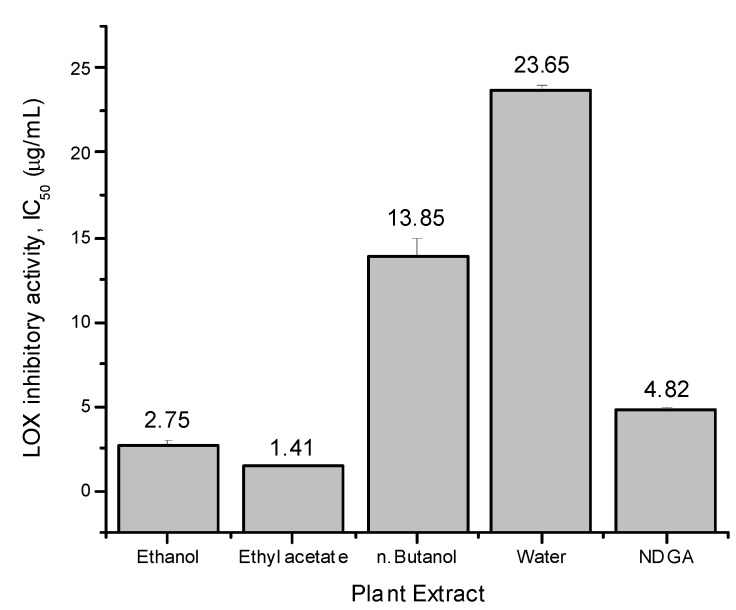
LOX inhibitory activities of ethanol extract and soluble fractions from *Leptadenia pyrotechnica* expressed as IC_50_ (µg/mL). Values are means ± SE of three experiments.

LOX catalyzes dioxygenation of polyunsaturated fatty acids to yield *cis,trans*-conjugated diene hydroperoxides. Results for LOX inhibitory activity (IC_50_) are shown graphically in Figure 1. The ethyl acetate fraction showed an improved ability to inhibit LOX activity (IC_50_ = 1.41 µg/mL) in relation to the ethanol crude extract (IC_50_ = 2.75 µg/mL). *n*-Butanol and water fractions showed much lower activities (IC_50_ = 13.85 µg/mL and 23.65 µg/mL, respectively). It is worth mentioning that both the ethyl acetate fraction and crude ethanol extract possessed significantly higher LOX inhibitory activity than that of an NDGA positive standard (IC_50_ = 4.82 µg/mL). Finally, LOX inhibition of all studied *Leptadenia pyrotechnica* fractions were higher than those for the common Oregon grape aqueous ethanolic extract (0.76 µg/mL) [9]. These results suggest that *Leptadenia pyrotechnica *has potentially high anti-inflammatory effect, which might be related to the polyphenolic content and antioxidant property of the extract.

### 2.4. Cytotoxicity

**Figure 2:**
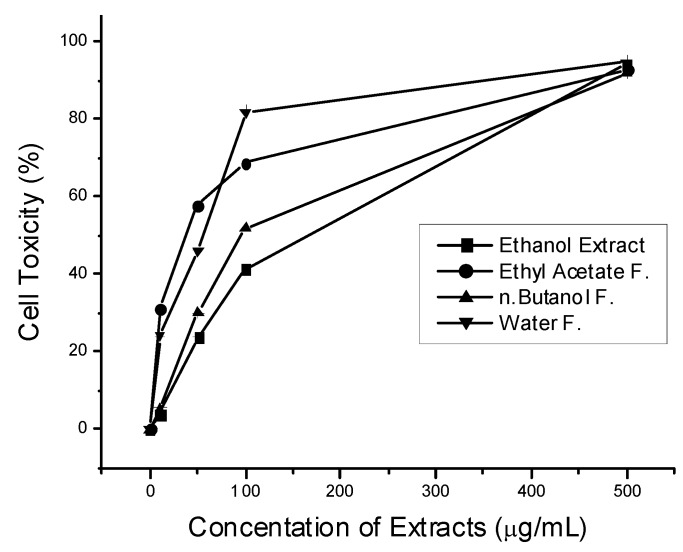
Cytotoxic effect of ethanol extract and partitioning fractions of *Leptadenia pyrotechnica* on MCF-7 breast cancer cells. Cell toxicity was measured by MTT assay. Data are means of percentage changes of control.

The inhibitory effect of cell growth was observed to be concentration dependent (Figure 2). The ethyl acetate fraction, once again, showed the strongest cytotoxic effect (IC_50_ = 43.16 µg/mL) followed by the water fraction (IC_50_ = 54.27 µg/mL) and butanol fraction (IC_50_ = 96.23 µg/mL), whereas the lowest effect was found in the crude ethanol extract (IC_50_ = 120.77 µg/mL). 

The fact that the ethyl acetate fraction exhibited the highest cytotoxic activity suggests that antioxidants play a major role in the cytotoxicity. However, the relatively higher than expected value for the water fraction (with the lowest TAC values) imply that phenolic compounds may not be the only compounds responsible for the cytotoxicity of *Leptadenia pyrotechnica *and suggest the possibility of the presence of other non-phenolic compounds with strong activity. This particular point needs further investigation.

## 3. Experimental

### 3.1. Chemicals

Ascorbic acid, ferric chloride, Folin-Ciocalteu reagent, dibutylhydroxytoluene (BHT), 2,4,6-tripyridyltriazine, gallic acid, sodium carbonate, 1,1-diphenyl-2-picrylhydrazyl (DPPH) and 2,4,6-tripyridyl triazine, 2,2-azino-bis(3-ethylbenzothiazoline-6-sulfonate) (ABTS), β-carotene (type I ≥ 93%), epicatechin (purity ≥ 90% HPLC), caffeic acid (≥98% HPLC) and quercetin-β-D-glucoside (≥90% HPLC) were obtained from Sigma Chemical Co. (St. Louis, MO, USA). Vanillic acid was obtained from Fluka (purum ≥97% HPLC). All other chemicals were obtained from common commercial suppliers.

### 3.2. Plant Materials

*Leptadenia pyrotechnica* was collected from the Al-Jimi area in the town of Al Ain in the southern part of the UAE. The harvested specimens were identified and voucher specimens were deposited at the Herbarium of the Biology Department, Faculty of Science, UAE University. 

### 3.3. Preparation of the Plant Extracts

A sample of the air-dried, ground aerial part of *Leptadenia pyrotechnica* (10 g) was extracted with 70% (v/v) aqueous ethanol (200 mL) and the mixture was macerated for 72 hours at 4 °C. The resulting mixture was then filtered and divided into two equal portions. The first portion was dried under reduced pressure in a rotary evaporator at 40 °C to give aqueous ethanol crude extract (referred to as crude extract). The other portion was concentrated under reduced pressure in a rotary evaporator and then suspended in water (50 mL). The mixture was extracted two times with *n*-hexane (2 × 100 mL) in a separatory funnel to remove fats. The residue was further extracted with ethyl acetate and *n*-butanol, respectively using a similar protocol. Each of the obtained fractions (ethyl acetate, *n*-butanol, along with the residual aqueous fraction) were dried, weighed, dissolved in DMSO (typically 25 mg/mL) and kept at −20 °C for further analysis. 

### 3.4. Determination of the Total Phenolic Content

Total phenolic content was determined by the method of Singleton *et al.* [17] using the Folin-Ciocalteu reagent. For a typical plant extract, a sample of the residue obtained from Section 3.3 (100 µL) was mixed with the Folin-Ciocalteu reagent (200 µL) and de-ionized water (2 mL) and incubated at room temperature for 3 min. Following incubation, a sample of 20% aqueous sodium carbonate (w/w, 1 mL) was added to the mixture. The total polyphenols were determined by measuring the absorbance of the resulting blue color at 765 nm with a Shimadzu UV-160 recording spectrophotometer after one hour of incubation at room temperature. Results were expressed in milligrams of gallic acid equivalent per g dry weight of plant material. Data presented are average of three measurements.

### 3.5. Estimation of Total Antioxidant Activity

#### 3.5.1. FRAP Assay

The FRAP assay is based on the reducing power of antioxidants by the reduction of the ferric ions to the ferrous ions, which form a blue colored ferrous-tripyridyltriazine complex according to the method described by Nenadis *et al.* [18]. The FRAP reagent was freshly prepared by mixing 2,4,6-tripyridyltriazine (10 mM, 1.0 mL) and ferric chloride (20 mM, 1.0 mL) in acetate buffer (0.25 M, 10 mL, pH 3.6). Plant extract sample (50 µL) was added to the FRAP reagent (3.0 mL). The tests were carried out in triplicate. The absorbance was measured at 593 nm after 8 min incubation at room temperature. A calibration curve of ascorbic acid was established for the quantitative determination of the antioxidant capacity of the plant extracts expressed as mmol ascorbic acid equivalent/g dry extract.

#### 3.5.2. ABTS Assay

This assay is based on the reduction of the blue-green 2,2-azino-bis(3-ethylbenzothiazoline-6-sulfonate) radical cation (ABTS·+) by antioxidants to its original colorless ABTS form. The ABTS·+ is decolorized by antioxidants according to their antioxidant capacities. A mixture of ABTS (10 mmol) and hydrogen peroxide (28.3 µmol) in acetic acid-sodium acetate buffer (30 mmol, pH 3.6, total volume of 2.0 mL) was rapidly mixed with the plant extract or standard compound (100 µL) in a test tube. Dibutyl hydroxytoluene (BHT) was used as a positive standard. The contents of the tube were swirled and allowed to stand for 6 min and the absorbance was measured at 660 nm [19]. Inhibition of free radical scavenging activity was calculated using the following equation:

% Inhibition =100 × (absorbance of the control − absorbance of the sample) / absorbance of the control. EC_50_ value (µg/mL) is the effective concentration at which ABTS·+ is scavenged by 50% and is determined graphically. A calibration curve of ascorbic acid was established, the antioxidant capacity of the plant extracts was then expressed as mmol ascorbic acid equivalent/g dry extract.

#### 3.5.3. DPPH· Radical Assay

The DPPH stable radical scavenging ability of plant extract was assessed according to a standard procedure [18]. Methanolic solution of DPPH radical (3.8 mL, 60 µg/mL) was rapidly mixed with the plant extract (200 µL) in a test tube. BHT was used as a positive standard. The contents of the tube were swirled and allowed to stand for 30 min and absorbance was measured at 517 nm. Inhibition of free radical scavenging activity was calculated using the following equation:

% Inhibition = 100 × (absorbance of the control − absorbance of the sample) / absorbance of the control. EC_50_ value (µg/mL) is the effective concentration at which DPPH· radicals are scavenged by 50% and is determined graphically. A calibration curve of ascorbic acid was established, the antioxidant capacity of the plant extracts was then expressed as mmol ascorbic acid equivalent/g dry extract.

#### 3.5.4. β-Carotene Bleaching Assay

The ability of the extracts to prevent bleaching of β-carotene was assessed according to the procedure of [11]. A sample of β-carotene solution (1.0 mL, 200 µg/mL in chloroform) was mixed with of linoleic acid (200 µL) and Tween 20 as emulsifier (200 µL). The mixture was evaporated to remove chloroform in a rotary evaporator at 40 °C. Deionized water (100 mL) was added slowly to form an emulsion. Aliquots of β-carotene/linoleic acid emulsion (3 mL each) were mixed in a series of test tubes with 200 µL of various plant concentrations. 200 µL of 50% of methanol in 3.0 mL of the above emulsion were used as control. BHT was used as positive standard. After incubation at 45 °C for 120 min, the absorbance of the samples, standards and control were measured at 470 nm. Inhibition of free radical scavenging activity was calculated using the following equation:

% Inhibition = 1 − (absorbance of the control at time zero − absorbance of the control after 120 min) / absorbance of the sample at time zero - absorbance of the sample 120 min) × 100. EC_50_ value (the effective concentration at which bleaching of β-carotene is prevented by 50% (µg/mL)) was determined graphically.

### 3.6. Lipoxygenase Inhibition Assay

Lipoxygenase (EC 1.13.11.12 type 1-B) (LOX) was assayed according to the method described by Wu *et al.* [20]. A mixture of a solution of sodium borate buffer (1 mL, 0.1 M, pH 8.8) and soybean LOX (10 µL, final conc. 8,000 U/mL) was incubated with plant extract sample (10 µL) in a 1 mL cuvette at room temperature for 5 min. The reaction was started by the addition of linolic acid substrate (10 µL, 10 mmol). The absorbance of the resulting mixture was measured at 234 nm as a function of time at a rate of one measurement/min (3 readings). Inhibition of LOX was calculated using the following equation:

% Inhibition = 100 × (absorbance of the control − absorbance of the sample) / absorbance of the control). The effective concentration (µg/mL) at which LOX activity is inhibited by 50% (IC_50_) was determined graphically. Nordihydroguaiaretic acid (NDGA) was used as a positive standard.

### 3.7. Cytotoxicity

The cytotoxicity of *Leptadenia* extracts on the MCF-7 human breast cancer cell line model was evaluated using the MTT (3-(4,5-dimethylthiazol-2-yl)-2,5-diphenylformazan) assay. Cells were grown in the DMEM medium (GIBCO-BRL) supplemented with 10% fetal calf serum (GIBCO-BRL, USA), 100 units/mL penicillin-streptomycin (GIBCO-BRL, USA) and non-essential amino acid (GIBCO-BRL, USA). The cells were maintained at 37 °C in 5% CO_2_ incubator. After reaching confluency, the cells were subcultured into 96 wells culture plates, allowed to grow for 1–2 days to a density of 2 × 10^4^ cells/well and treated with different concentrations of *Leptadenia pyrotechnica* extracts (final concentrations 10–500 µg/mL in 0.05% DMSO) for 24 hours. MTT solution with DMSO served as a negative control and with SDS as a positive control. The MTT [3-(4, 5-dimethylthiazol-2-yl)-2-5-diphenyltetrazolium bromide] assay was performed according to the manufacturer’s (Promega, WI, USA) instructions to examine the cytotoxic effect of these compounds. Briefly, 20 μL of MTT reagent (CellTiter 96-Aquous non-radioactive cell proliferation Assay, Promega, WI, USA) was added to the each well, and incubated the cells for one to two hours. This assay is a colorimetric assay for cell viability based on the cellular cleavage of the yellow tetrazolium salt, MTT, into the purple formazan crystals that is soluble in cell culture medium and is measured at 490 nm directly in 96-well assay plates. Absorbance is directly proportional to the number of the living cells in culture. Viability was calculated as a percentage of the control cells. All experiments were carried out in triplicates. The IC_50_ value was defined as the concentration of plant extract necessary to inhibit the growth to 50% of the control.

### 3.8. Identification of Main Polyphenolic Compounds

HPLC analyses were performed according to the method of Abad-Garcia *et al.* [21] with an Agilent 1200 LC system consisting of degasser, quaternary pump (G1311A), auto sampler (G1329A), column heater (G1316A) and diode array detector (DAD) (G1315C). A reversed phase C18 (4.6 mm × 250 mm × 5 µm) column (Waters Corporation, Milford, MA, USA) was used. The mobile phase used in this study was a gradient of two solvents: solvent A (acetic acid-water, 0.5:99.5, v/v) and B (methanol). The gradient profile was as follows: 0–2 min, 0% B isocratic; 2–6 min, linear gradient from 0% to 15% B; 6–12 min, 15% B isocratic; 12–17 min, linear gradient from 15% to 20% B; 17–35 min, 20% B isocratic; 35–90 min, linear gradient from 20% to 35% B; 90–136 min, 35% B isocratic. After each run, the system was washed and reconditioned for 10 min before analysis of next sample. The flow rate was 0.8 mL/min and injection volume was 50 µL. A series of individual standard solutions of polyphenolic compounds (each 1.0 mg/mL) dissolved initially in minimal amount of DMSO and diluted with aqueous methanol (50:50 v/v) was used to generate calibration curves. Polyphenolic compounds were identified and quantified in the samples by using retention times and calibration curves, respectively. The detection was carried out at 254, 280, 320 and 370 nm.

### 3.9. Statistical Analysis

All analyses were repeated three times to ensure accuracy. Reported data are expressed as means ± SEM. Correlation analysis of antioxidants *versus* the total phenolic content was carried out using the regression analysis with SPSS version 10 statistical program (SPSS Inc., Chicago, IL, USA). When significant differences were detected by using ANOVA, analysis of differences between the means of the measurements was performed by using Turkey's multiple comparison test. Significance was determined at p < 0.05 level.

## 4. Conclusions

In conclusion, biological investigation *Leptadenia pyrotechnica* extract demonstrated promising antioxidant, anti-inflammatory and anti-cancer properties mainly for the crude extract and the ethyl acetate fraction. These biological activities could be presumably ascribed in part to the polyphenolic as well as other constituents of these fractions. Therefore, this study provides the biochemical rationale for further chemical analysis as well as animal and clinical studies running underway in our labs.
